# Anisakiasis in Italy: Analysis of hospital discharge records in the years 2005-2015

**DOI:** 10.1371/journal.pone.0208772

**Published:** 2018-12-11

**Authors:** Serena Cavallero, Agnese Martini, Giuseppe Migliara, Corrado De Vito, Sergio Iavicoli, Stefano D’Amelio

**Affiliations:** 1 Department of Public Health and Infectious Diseases, Sapienza University of Rome, Rome, Italy; 2 Italian Workers' Compensation Authority-Department of Occupational and Environmental Medicine, Epidemiology and Hygiene, Rome, Italy; Universita degli Studi di Camerino, ITALY

## Abstract

**Background:**

Anisakiasis is a fish-borne zoonosis caused by the ingestion of marine food infected with *Anisakis* third-stage larvae, widespread marine parasitic nematodes. Gastrointestinal and/or allergic clinical signs and symptoms are not specific. While frequently reported in countries with large raw fish consumption as Japan, the global prevalence of anisakiasis may be severely underestimated due to limitations of available diagnostic tools and to diverse clinical manifestations. Recently, infective larvae were found in the same localization with gastrointestinal tumors. The occurrence of allergic exacerbation upon secondary exposure and the possible occupational exposure, highlight the need to increase scientific evidences on anisakiasis.

**Methods:**

We performed a retrospective descriptive study using analysis of Hospital Discharge Records (HDRs) from 2005 to 2015 in Italy, with particular attention to allergic manifestations. Descriptive statistics and multivariate analyses were performed using backward step-wise logistic regression models to assess spatial distribution and temporal trend as well as the variables independently associated with the allergic clinical signs and symptoms in Italian cases of anisakiasis.

**Results:**

HDRs reporting the ICD-9 code for anisakiasis were retrieved (370), with a higher number of cases reported from central and southern regions, with particular regard to populations inhabiting the coastal territories. Around 40% of patients presented allergic manifestations and half of them showed serious allergic reactions. The multivariate analyses showed an independent association between allergic manifestations and features as living in southern regions and female gender, while anaphylactic episodes was independently associated only with female gender.

**Conclusion:**

The present study is the first attempt to a better understanding of the epidemiological picture of anisakiasis in Italy, mining official data. A common strategy on data collection, monitoring and reporting would favor a more accurate epidemiological scenario in Italy, since the report of the diseases is not mandatory.

## Introduction

Anisakiasis is a fish-borne zoonotic infection caused by third-stage larvae of *Anisakis*, parasitic anisakid nematodes with a cosmopolitan distribution, depending on aquatic hosts to successfully complete their life-cycle [1]. Definitive hosts are cetaceans and less frequently pinnipeds, which harbor the adult nematodes, while intermediate and paratenic hosts infected by larval stages are crustaceans and fishes and/or cephalopods, respectively. The term anisakidosis refers to the same illness caused by species belonging to members of the family Anisakidae, thus including species of the *Pseudoterranova decipiens* complex, which infect mostly pinnipeds as definitive hosts.

The occurrence of larval nematodes in fish fillets, squids or fish products is of medical and economic concern: beside the effects on consumers’ perception and marketability, larvae cause anisakiasis when infected raw or undercooked seafood is ingested, provoking a mild to severe disease. Clinical signs include gastrointestinal and/or allergic symptoms as epigastralgia, diarrhea, nausea, abdominal pain and/or urticaria, rhinitis, bronco-constriction.

Anisakiasis can be classified into gastric (GA) and intestinal (IA) or ectopic/extra-gastrointestinal depending on the location of the larva; in acute/chronic and severe/moderate by the entity of symptoms and in invasive and not invasive depending on larval behavior [2]. Another form of the disease is known as Gastro-Allergic Anisakiasis (GAA), caused by a combination of abdominal discomfort and IgE-mediated allergic reactions [3]. After initial manifestations, anisakiasis may become chronic if the immune response does not eliminate the worm, and subsequent sensitizations to *Anisakis*-derived allergens possibly trigger allergic exacerbation upon secondary exposure [4]. Allergic reactions are also related to a limited exposure to larvae or parasitic allergens intended as residues in food, by contact or inhalation [5].

Firstly reported in the Netherlands [6], anisakiasis is of public health relevance in eastern countries such as Japan where raw fish is largely consumed, but it has gained a growing attention in other western countries too, as United Kingdom, France, Spain, Croatia and Italy where human cases are increasingly reported [7–8]. Nevertheless, Spain appears to have the highest reported incidence of anisakiasis in Europe, and “boquerones en vinaigre” (marinated anchovies) are recognized as the main food vehicle [9]. Several local traditional fish preparations are considered to be of high risk for anisakiasis: Japanese sushi and sashimi, Spanish and Italian marinated anchovies, Scandinavian gravlax, Dutch salted and marinated herring, South American ceviche. It is worth to underline that most of the South American reported cases are caused by *Pseudoterranova* spp. [10], which has been recently observed also in Italy [11] and in France [12].

Anisakids are considered the only fish-borne parasites able to elicit an allergic response in humans and a predominant biological hazard associated to seafood, due to the high prevalence recorded in the main fishery species worldwide [13–17].

Diagnostic approach useful to detect and identify etiological agents of anisakidosis are mainly endoscopic [18], serological [19–20], by imaging tools [21] and by molecular approach [22–23].

Recently, *Anisakis* spp. have been observed in the same localization with gastro-intestinal tumors [24–26], renewing the attention to potential consequences of infection. Due to intrinsic limitations of currently available diagnostic tools and to a plethora of diverse and aspecific symptoms, the global prevalence of gastrointestinal and allergic anisakiasis is likely to be severely underestimated.

With the aim to provide an assessment of the epidemiology of anisakiasis in Italy based on official data, we describe for the first time anisakiasis related hospitalizations between 2005 and 2015, using HDRs (Hospital Discharged Records), in terms of time, geographical distribution, and disease related individual characteristics.

## Methods

### Data analysis

We performed a retrospective study based on data from patients collected from Hospital Discharge Records (HDRs), obtained from the official databases of the Ministry of Health, during a 11-year period: January 1, 2005 to December 31, 2015. The International Classification of Diseases, Ninth Revision, Clinical Modification [27] (ICD-9-CM version employed during the study period) for encoding diagnosis and procedures contained in the HDRs has been adopted as the standard (Center for Disease Control and Prevention, 2010). For each entry, we collected sociodemographic and clinical data such as gender, age, born and domicile code, educational level, region and province (with particular attention to distance from the seaside: residence less then 25km from the seaside is considered “coastal” while over 25km is considered “hinterland”), primary and secondary diagnosis codes, surgical or medical procedure codes, duration of hospitalization and mortality data.

We estimated the average number of hospitalizations per year and regions in order to assess temporal and geographical patterns; official population estimations obtained from the Italian Institute of Statistics (ISTAT) regarding the eleven-year interval were considered as population at risk to estimate incidence of anisakiasis.

All cases marked as principal diagnosis (PRD) are those reporting the code 127.1 (Anisakiasis) as first diagnosis at hospital acceptance. Other co-diagnosis of interest related to anisakiasis were selected and furtherly explored in all remaining cases. The “anisakiasis-related symptoms”, as defined in previously described cases, were classified mainly as allergic and gastrointestinal (Table 1).

**Table 1:** Symptoms and clinical manifestation related to anisakiasis.

Allergic	Gastrointestinal
Erythema toxicum	Pain with not-specified location
Allergic contact dermatitis associated with food	Heartburn
Other atopic dermatitis and related conditions	Gastroenteritis and colitis—non infective
Other psoriasis	Acute Gastritis
Allergic purpura	Unspecified intestinal obstruction
angioneurotic edema	Other specified intestinal obstruction
Intrinsic non allergic asthma	Acute duodenal ulcer with perforation, without mention of obstruction
Extrinsic allergic asthma with acute exacerbation	Diverticulosis of colon (without bleeding losses)
Allergic rhinitis due to food	Other suppurative peritonitis
Unspecified asthma	Pirosis
Cholinergic urticaria	Acute gastric ulcer with hemorrhage
Other specified urticaria	Other suppurative peritonitis
Allergy, unspecified, not elsewhere classified	Regional enteritis of unspecified site
Anaphylactic reaction due to unspecified food	Abdominal epigrastric pain
Shock, not elsewhere classified	Right upper quadrant abdominal rigidity
Eosinophilia	Irritable bowel syndrome
Acute bronchospasm	Unspecified gastritis and gastroduodenitis, without bleeding
Sicca syndrome [Sjögren]	Left lower quadrant abdominal pain
	Benign neoplasm of large intestine
	Diarrhea
	Dyspepsia and other specified disorders of function of stomach
	Chronic atrophic gastritis without bleeding
	Regional enteritis of large intestine
	Ulcerative pancolitis
	Intestinal malabsorption, unspecified
	Intestinal or peritoneal adhesions with obstruction
	Regional enteritis of small intestine
	Non-specific mesenteric lymphadenitis
**Others**	
Cephalalgy*	
Benign hypertensive heart disease with congestive heart failure*	
Precordial pain*	

List of symptoms and clinical manifestation related to anisakiasis reported in the dataset.

*Information not directly included in the two main categories.

Descriptive statistics for all variables was estimated. To assess the association with allergic symptoms and, specifically, with anaphylactic shock, univariate analysis was performed using the Chi2 test, or the exact Fisher test when appropriate, for dichotomous and categorical variables and the Wilcoxon-Mann-Whitney test for continuous non-normal variables. Stepwise logistic multiple regression models were built to identify possible predictors of allergic symptoms and anaphylactic shock. Variables were included in the models when the *p* value at the appropriate univariate test were lower than 0.25. Age and gender were always included in the models for their clinical significance. The other variables included in the models were: duration of hospitalization, living in the coast or in the hinterland, living in the southern regions, the reason for hospital admission (only for the severe allergic symptoms model) and the educational level. Variables were maintained in the model for a *p* value lower than 0.05. A *p* value lower than 0.05 was considered statistically significant. All analyses were performed with STATA 15 (StataCorp LLC, 4905 Lakeway Drive, College Station, Texas, USA).

### Ethic statement

Hospital Discharge Records (HDRs) were provided from the official databases of the Ministry of Health, following the current Italian legislation (Registry 1.9.b/3—Ministry of Health—General Directorate for Health Planning—database HDRs). The availability of these databases do not require approval from ethical committees.

In compliance with the current Italian legislation on privacy, the publication and or dissemination of HDR's data and their processing must take place only in aggregate form, without any reference to patients’ personal information. Anonymous personal data are available according to the current privacy regulations (L.675/96) (Gazzetta Ufficiale, 1997).

## Results

### Spatial and temporal trend in Italy

A total of 370 HDRs reporting the specific ICD-9 code for Anisakiasis (127.1) were retrieved. Spatial and temporal distribution of hospitalization related to the disease showed central and southern regions with the highest number of cases reported (more than 90%). In fact, almost half of HDRs originated from Apulia, south of Italy (51.1%), followed by four central regions as Abruzzo (11.4%), Marche (8.1%), Campania (6.2%) and Latium (5.7%). Only 6 out of 370 patients had foreign nationality (two from the north, one from a central region and three from the south). Considering the coastal or inland territories, 80.3% of patients were living close to the coast, while 19.7% lived in the hinterland. At national level, the annual hospitalization was ranging from 1.3 to 15.5 out of 100.000, with the highest number of new cases in 2011 (67 of which diagnosed in Apulia). Hospitalization trends from those regions with higher rates revealed different temporal patterns, with highest number of case reported in 2010, 2011 and 2014 for Latium, Apulia and Abruzzo, respectively.

### Clinical characteristics of anisakiasis related hospitalizations

Patients were 48.9% men and 51.1% women, with a mean age of 46.7 (sd ±17.4, range 1–90) and the highest frequency found in the 40–49 age group with 21.4% of cases. The mean length of hospital stay was 5.7 days (sd ±5.9, range 1–71); 62.7% of patients showed a lower educational level, 29% had high school graduation degree and a residual 8% had a university degree (Table 2).

**Table 2:** Descriptive Statistics from anisakiasis HDRs.

Descriptive Statistics from Anisakiasis HDRs		
Features of interest	Number	%
Gender	Female n = 189 Male n = 181	51.1% 48.9%
Age	mean 46.7 (sd± 17.4, range 1–90)	
Length of hospital stay	mean 5.7 days (sd ±5.9, range 1–71)	
Educational level	low (primary and secondary school) n = 180 high school n = 85 university n = 22	62.7% 29.6% 7.7%
Regions	North n = 24 Central n = 100 South n = 246	6.5% 27% 66.5%
Residence	Coastal n = 297 Hinterland n = 73	80.3% 19.7%

Characteristics of interest selected from HDRs of anisakiasis cases reported in Italy from 2005 to 2015.

Around 32% of cases have been detected at their admission and are ranked as principal diagnosis “PRD”; for the remaining cases, 52.2% showed symptoms and/or clinical manifestations already reported in literature as related to anisakiasis, mainly involving the digestive tract or allergy. Among these, 61.1% showed allergies, and 45.1% exhibited anaphylactic reactions. In the eleven years period, the highest number of serious allergic related cases is reported between 2011 and 2012. The trend of the abovementioned features is shown in Table 3.

**Table 3:** Temporal distribution of anisakiasis cases (according to years and diagnosis).

Year	PRD	Gastro -intestinal	Allergic	Others
2005	17.6%	35.3%	5.9%	41.2%
2006	50.0%	16.7%	16.7%	16.7%
2007	25.0%	12.5%	37.5%	25.0%
2008	44.4%	55.6%	0.0%	0.0%
2009	56.3%	31.3%	6.3%	6.3%
2010	36.9%	21.5%	30.8%	10.8%
2011	18.7%	17.6%	49.5%	14.3%
2012	26.9%	6.0%	53.7%	13.4%
2013	41.9%	19.4%	22.6%	16.1%
2014	44.8%	34.5%	6.9%	13.8%
2015	47.6%	28.6%	4.8%	19.0%

Temporal distribution of anisakiasis cases (according to years), classified using "principal diagnosis" as feature (PRD), with symptoms and clinical signs related (gastro-intestinal and allergic) and not related to anisakiasis (others).

Surgical procedures after diagnosis included ulcer sutures, partial intestinal resection and gastrectomy, while medical diagnostics procedures included endoscopy, colonoscopy, biopsy, imaging (TC scan, NMR, X-rays, ultrasound and scintigraphy).

The univariate analysis showed that allergic anisakiasis is significantly associated with living on the coast (coast 144/285 vs hinterland 18/85, p<0.001), and in the southern regions of Italy (South 147/277 vs North-Central 14/82, p<0.001). Regarding the demographic features of the patients, allergic symptoms are significantly associated with female gender (female 100/189 vs male 62/181, p<0.001). A significantly different distribution of the allergic symptoms was detected among educational levels (p<0.001), with the higher frequency among people with primary school (68/111) and high school diplomas (46/85). A statistically significant higher frequency (p<0,001) was also found among “persons who presented themselves to the hospital without a doctor request” (100/190). Finally, the days of hospitalization were significantly fewer for patients with allergic symptoms than for other patients (Median 3, IQR 2–5 vs Median 5, IQR 3–9, p<0.001). The same features as above were found to be positively associated with patients with anaphylactic shock (see Table 4).

**Table 4:** Univariate—allergic symptoms and clinical manifestations.

Features of interest	Number	*p*-value
**a. Allergic symptoms and clinical manifestations**		
Gender	Female n = 100/189 Male n = 62/181	<0.001
Reason for hospital admission	doctor indication scheduled recovery without doctor indication transfer	0.001
Educational level	low (primary and secondary school) n = 88/180 high school n = 46/85 university n = 10/22	0.001
Regions	South *vs* Central-North	<0.001
Residence	Coastal vs Hinterland	<0.001
**b. Anaphylactic shock**		
Gender	Female n = 55/189 Male n = 32/181	0.01
Reason for hospital admission	medical referral scheduled recovery autonomous referral transfer	<0.001
Educational level	low (primary and secondary school) n = 88/180 high school n = 46/85 university n = 10/22	0.02
Regions	South *vs* Central-North	<0.001
Residence	Coastal vs Hinterland	<0.001

Univariate analysis of the HDRs characteristics of interest and association with a. allergy, specifically with b. anaphylactic shock (Chi2 test, exact Fisher test, Wilcoxon-Mann-Whitney test; p-value).

At the multivariate analysis, the variables independently associated with allergic symptoms were female gender (OR: 2.26; 95% CI: 1.29–3.98), living in a southern region (OR: 12.91; 95% CI: 4.34–38.34) and the primary school diploma (OR: 1.91; 95% CI: 1.08–3.39). Moreover, the period of hospitalization was significantly shorter for these patients (OR: 0.80; 95% CI: 0.73–0.88).

The female gender was also a predictor of anaphylactic shock (OR: 5.10; 95% CI: 1.76–14.8), as well as the older age (OR: 1.04; 95% CI: 1.01–1.08), the primary school diploma (OR: 7.07; 95% CI: 1.89–26.45), the high school diploma (OR: 5.78; 95% CI: 1.38–24.19) and the “persons who presented themselves to the hospital without a doctor request” (OR: 154; 95% CI: 15.60–1521.36). This group of patients were also significantly associated with shorter periods of hospitalization (OR: 0.36; 95%CI: 0.25–0.53). See Table 5 for the results of multivariate analyses.

**Table 5:** Multivariate—allergic symptoms and clinical manifestations.

Variable	OR	95%CI	*p*-value
**a. Mild to severe allergic reactions**			
Southern regions	12.91	4.34–38.34	<0.001
Length of hospital stay	0.80	0.73–0.88	<0.001
Female gender	2.26	1.08-3-39	0.026
low educational level	1.91	1.29–3.97	0.004
**b. Anaphylactic shock**			
Age	1.04	1.01–1.07	0.008
Female gender	5.10	1.75–14.82	0.003
Primary School Diploma (vs No Study Title)	7.06	1.88–26.45	0.004
High School Diploma (vs No Study Title)	5.78	1.38–24.18	0.016
Autonomous Referral (vs Medical Referral)	279	28.57–2738.95	<0.001
Length of hospital stay	0.36	0.24–0.53	<0.001

Multivariate logistic regression model for potential predictors of the allergic reactions in anisakiasis (a. mild to severe reactions b. anaphylactic shock). OR: odds ratio; SE: 95% confidence interval; *p*-value.

## Discussion

The present study is the first attempt to analyse anisakiasis in Italy with the support of hospital discharge records as source of information, to be critically assessed in the light of epidemiological and clinical published data. Unfortunately, HDRs do not include any mention on laboratory diagnostic methodologies, an information potentially useful to infer the reliability of the cases reported as anisakiasis; moreover, HDRs correspond only to hospitalized cases thus depicting an underestimated incidence of anisakiasis in Italy as suggested also by a very recent study conducted in Spain using the same approach [28].

The obtained data describes a potential association between anisakiasis showing allergic features with i) female gender, ii) living in coastal areas, iii) in particular from southern regions and with iv) low-medium educational level.

A recent retrospective study aimed to analyse Italian cases reported in literature showed the same regions (Abruzzo, Latium and Apulia) as those mainly involved in anisakiasis and *A*. *pegreffii* as the only species identified in molecularly diagnosed cases [29].

Similar attempts aimed to better understand anisakiasis in European countries have been recently published [30]: in a national retrospective survey of anisakidosis in France, an apparent decrease in clinical cases and a potential emergence of allergy were reported. On a total of 37 cases, around 50% indicated anisakidosis as the main diagnosis of hospitalization. Accordingly, there was a significant predominance of women among reported cases. Similarly, we observed a significant association between allergic reactions and female gender. Although the number of cases of anisakiasis is very similar in males and females, women seems to be more prone to allergic/anaphylactic reactions. It should be noticed that female sex steroid hormones are believed to play a role in allergic reactions [31].

Reports on the admission to the emergency departments of gastric and small intestinal anisakiasis cases in Japan highlighted the importance of an early and accurate diagnosis, based also on the history of raw food ingestion, evaluation of symptoms timing and classic imaging findings [32]. As suggested, anisakiasis shows non-specific symptoms and clinical signs, so anamnestic analysis is crucial for the onset of a correct diagnostic *iter*. In the present study, a principal diagnosis classified as “Anisakiasis” was associated only to a relatively low percentage of cases (119/370), thus suggesting a possible difficulty in disease identification or a scarce awareness by healthcare operators. A relevant proportion of cases (23%) was characterized by serious allergic reactions. These cases dramatically increased around 2011 and in particular in Apulia region, probably due to a greater awareness of scientific and medical communities and consumers for anisakiasis. Subsequently, a specific regulation of the Italian Ministry of Health, based on mandatory indication for consumers to deep-freeze those fish products intended for raw consumption, was applied in 2013, with the aim to mitigate risk of anisakiasis and to inform consumers, probably contributing to a higher awareness and prevention. As *Anisakis* is a widespread species being naturally part of the marine biodiversity, the best control strategies are based on monitoring source of infection and providing information to consumers and food operators. The European Food Safety Authority (EFSA) released a scientific opinion on zoosanitary parasite control of fishery products for human consumption. They indicated protection and prevention as priorities and recommended a continuous research in parasites of public health importance in fishery products^14^. The most recent epidemiological data on the presence of infective larval stages in fish and fish products intended for human consumption reported a positive association between the number of larvae recovered and the size of fish, and the recovery of larvae in fillets with time passed by fish death and temperature of storage [33]. Differences in epidemiological parameters reported in literature could be attributed to different variables including different fishing areas or differences in fishing seasons and, therefore, differences in fish body size [34]. The most recent reports of anisakid larvae from fish collected in the Mediterranean basin described a high levels of infection in two commercially important fish species as *Merluccius merluccius* and *Engraulis encrasicolus* [35–36]. As anchovies are generally consumed in different raw or marinated dish preparations, their consumption represent a major risk for anisakiasis [9]. Levels of infection with *A*. *pegreffii* in anchovies significantly varied between the selected fishing areas: fish from the Central and South Adriatic Sea showed the highest levels of infection while those from Southern Sicily, Ionian and Alboran Seas were uninfected [35]. Regarding the European hake *M*. *merluccius*, high levels of infection with *A*. *pegreffii* were recorded from the Adriatic/Ionian Sea [36].

It is important to underline that even if most of larvae were recovered from fish visceral cavity and a smaller amount from fillets, the potential risk for anisakiasis is still present because a single larva is sufficient to the onset of clinical symptoms and disease. Moreover, a recent investigation on different kinds of ready-to-eat products made of anchovies, sold in Italian supermarkets, showed that semi-preserved products heavily contaminated reached the market, representing a potential risk related to allergic reactions in sensitized individuals [37]. Consequently, a continuous monitoring of anisakid infections in fishes destined to human consumption appears needed, particularly regarding specific target species.

Interesting aspects not yet fully understood are those related to allergy: dynamics and schemes of allergic reactions are still obscure [38], renewing an open debate on the necessity of larvae being alive to induce allergic reactions such as urticaria or anaphylaxis. Together, major antigens useful to develop reliable tools for serological diagnosis are still needed. A recent study carried out by AAITO-IFIACI *Anisakis* Consortium [39] suggested that *Anisakis* hypersensitivity and allergy is mainly a matter of dietary habits. During 2010, 34 Italian allergy centers were screened by the mean of specific interview and skin prick test (SPT) with *Anisakis* extract. Among over 10K people included, 4.5% scored positive and 14% of those sensitized had a history of allergy to *Anisakis*. Marinated anchovies were the most frequent mentioned cause of allergic reactions and sensitization rate showed marked geographic differences along the Adriatic and Tyrrhenian coasts, where homemade marinated anchovies are an age-old tradition. In inland towns of northern Italy, the prevalence was directly related to the number of inhabitants.

## Limitations and conclusions

In conclusion, data mining from HDR provides evidences that should be considered with caution, considering the possibility of errors in diagnosis, reporting, classification, not to mention possible typo-errors by administrative officers. Moreover, our study included cases of anisakiasis with hospitalization, which is not equivalent to the true anisakiasis incidence in the population. Important individual data such as occupation and/or other possible related risk factors are missing. However, the results here obtained describe a scenario that is substantially in agreement with the high prevalence of *Anisakis* larvae in anchovies caught off coastal zones (i.e. coastal areas of central and southern Adriatic Sea [35]), with the high incidence of anisakiasis and the largely frequent habits of consuming raw marinated anchovies in such territories.
